# Effect of ethnicity on vinorelbine pharmacokinetics: a population pharmacokinetics analysis

**DOI:** 10.1007/s00280-019-03872-9

**Published:** 2019-05-27

**Authors:** Aurélie Pétain, Dafang Zhong, Xiaoyan Chen, Zhang Li, Shao Zhimin, Jiang Zefei, Grégoire Zorza, Pierre Ferré

**Affiliations:** 1 Institut de Recherche Pierre Fabre, 3 Avenue Hubert Curien, 31035 Toulouse, France; 2 Shanghai Institute of Materia Medica, Chinese Academy of Science, Shanghai, People’s Republic of China; 3 Sun Yat-Sen University Cancer Center, Guangzhou, People’s Republic of China; 4 Fudan University Cancer Hospital, Shanghai, People’s Republic of China; 5 307 Hospital of PLA, Beijing, People’s Republic of China

**Keywords:** Vinorelbine, Non-small-cell lung cancer, Breast cancer, China, Pharmacokinetics

## Abstract

**Background:**

Pharmacokinetics of vinorelbine is mainly known from studies conducted in European patients. Interethnic differences in drug disposition may, however, induce interethnic variation in drug exposure. This paper aimed to evaluate the effect of ethnicity on the bioavailability and clearance of oral and intravenous vinorelbine.

**Methods:**

Oral and intravenous vinorelbine pharmacokinetics data in Asian patients were pooled from two-phase II studies of patients with non-small-cell lung cancer or advanced breast cancer in China. Blood vinorelbine and its active metabolite, 4′-*O*-deacetylvinorelbine, were quantified using liquid chromatography–tandem mass spectrometry. Bayesian pharmacokinetic parameters were calculated and vinorelbine monotherapy results (intravenous 25 mg/m^2^; oral 60 mg/m^2^) of the Asian data set were compared to a reference European data set (intravenous 30 mg/m^2^; oral 80 mg/m^2^). Subsequently, a population pharmacokinetics analysis was conducted in a combined cohort (Asian data set + historical vinorelbine pharmacokinetics database) to investigate for a potential effect of ethnicity.

**Results:**

Pharmacokinetics data from the Asian data set (oral: *n* = 47; intravenous: *n* = 34) was compared to the European reference data set (oral: *n* = 48; intravenous: *n* = 48). Mean apparent clearance of oral vinorelbine and mean absolute clearance of intravenous vinorelbine was comparable between the Asian and reference European data set. A population pharmacokinetic analysis (oral: *n* = 222; intravenous: *n* = 111) demonstrated no influence of ethnicity on oral and intravenous vinorelbine bioavailability and clearance.

**Conclusion:**

Vinorelbine pharmacokinetics were found to be comparable between Asian and European patients. No relevant influence of ethnicity on vinorelbine bioavailability and clearance for oral and intravenous routes of administration was observed.

## Introduction

Vinorelbine is a vinca alkaloid that is widely used for the treatment of advanced or metastatic non-small-cell lung cancer (NSCLC) and advanced breast cancer (ABC). Vinorelbine has been marketed in France since 1989 and China since 1993 as an aqueous solution for intravenous administration (Navelbine^®^, 10 mg/ml). A new oral formulation of vinorelbine has been developed as a line extension of the intravenous formulation for the same indications. Oral vinorelbine was developed as soft gelatin capsules and became available for clinical use in Europe since 2001, and more recently in China in 2014 [1].

During its clinical development, the pharmacokinetics of intravenous vinorelbine have been investigated and documented in a series of phase I [2, 3] and phase II studies [4]. The pharmacokinetics of intravenous vinorelbine is characterized by a large volume of distribution (between 70–75.61 l/kg) [5, 6], reversible binding to blood platelets (> 70%) [5, 7], and low protein binding (13%) [8]. Pharmacokinetic characteristics of oral vinorelbine were obtained from traditional pharmacokinetic analysis within each clinical study, and from population pharmacokinetic analysis conducted on pooled data sets from clinical studies [9, 10]. Oral vinorelbine is rapidly absorbed with an absolute bioavailability of approximately 40% [11]. Drug exposure is not influenced by food intake [12], and inter-individual variability is the same as that for the intravenous formulation [10, 13]. Reproducible drug exposures were observed over successive courses, and a dose-proportional increase in exposures was established [10, 14]. The pharmacokinetics of oral vinorelbine has been investigated in special sub-populations such as the elderly [15] and liver-impaired patients [16]. No dose adjustments of vinorelbine are required for elderly patients or patients with mild-to-moderate liver impairment [15, 16].

The equivalence between oral and intravenous doses required to achieve comparable blood exposure was established at 60 and 80 mg/m^2^ oral vinorelbine for 25 and 30 mg/m^2^ intravenous vinorelbine, respectively [11]. In a randomized phase II study, both formulations showed comparable activity and a qualitatively similar safety profile [4].

The metabolism pathway of vinorelbine primarily involves liver CYP3A4 enzymes to form inactive metabolites, except for, 4′-*O*-deacetylvinorelbine, the only active metabolite likely formed by carboxyl-esterases [17]. For both oral and intravenous administrations, bile is the major route of elimination for vinorelbine and its metabolites. Less than 10% of vinorelbine is eliminated via the urine, which mainly involves the parent compound [18].

Clinical studies of oral and intravenous vinorelbine have been mostly conducted in European patients. However, two-phase II studies have been completed in China to document oral and intravenous vinorelbine pharmacokinetics in Asian patients. One study was conducted in patients with ABC, and the other in patients with NSCLC. In both studies, patients were randomized to receive either intravenous or oral vinorelbine and the pharmacokinetic profile of vinorelbine was assessed. The objective of the present paper was to compare the pharmacokinetics of vinorelbine in Asian patients from pooled data of the two-phase II studies in China with those of European patients reported in Bourgeois et al. [11]. A population pharmacokinetics analysis was then conducted to further evaluate the effect of ethnicity on the bioavailability and clearance of oral and intravenous vinorelbine.

## Methods

### Description of clinical studies

The present study is based on pooled pharmacokinetic data obtained from two prospective, multicenter, open-label, randomized phase II trials conducted in ABC and NSCLC patients in China. Both studies were conducted in accordance with the Declaration of Helsinki and the International Conference on Harmonization Good Clinical Practice guidelines, local laws, and applicable regulatory requirements. The study protocols and their related documents (including the patient information and informed consent form) were approved by the local Ethics Committees and Competent Authority. All patients provided written informed consent for participation in the studies.

For the NSCLC study, key inclusion criteria were as follows: aged between 18 and 75 years (inclusive); men or non-pregnant, non-lactating women; cytologically or histologically confirmed diagnosis of NSCLC; stage IIIB, IV, or inoperable relapsed disease of any stage; and not previously treated with chemotherapy or immunotherapy. For the ABC study, key inclusion criteria were as follows: aged between 18 and 70 years (inclusive); non-pregnant, non-lactating women; and histologically confirmed adenocarcinoma of the breast. For both studies, eligible patients should have adequate bone marrow, hepatic and renal functions, and those over 65 years should not have more than three relevant co-morbidities that affect cardiac, pulmonary, liver, or renal functions.

Common key exclusion criteria for both studies were as follows: concomitant treatment with any other anticancer drugs; presence of malabsorption syndrome or disease significantly affecting gastro-intestinal function or major resection of the stomach or proximal small bowel that could affect absorption of oral vinorelbine; known hypersensitivity to drugs with similar chemical structures of study drugs; concomitant treatment with corticosteroids except chronic treatment lasting more than 1 month, given at low doses (≤ 20 mg/day of methylprednisolone or equivalent).

### Treatments

Patients in both studies were randomized to receive either 60 mg/m^2^ oral or 25 mg/m^2^ intravenous vinorelbine during the first treatment cycle on days 1 and on days 8. For oral administration, vinorelbine supplied as 20 mg and 30 mg capsules (Navelbine^®^ Oral) were administered after meals in the presence of a healthcare professional. Patients were instructed not to chew or suck the capsules and to ingest the capsules rapidly in whole. In case of vomiting after oral administration, the study drug was not replaced. For intravenous administration, vinorelbine supplied as 10 mg/ml vials (Navelbine^®^ i.v.) was reconstituted in normal saline aqueous solution and infused intravenously over 6–10 min after thorough dilution and under close supervision. In both studies, patients received vinorelbine alone on day 8 or in combination with capecitabine (ABC) or cisplatin (NSCLC), on day 1. Only pharmacokinetic data generated with vinorelbine given alone were used for ethnicity analysis in the current study.

### Pharmacokinetic assessment

Blood samples were collected over 24 h during the first and second administrations of vinorelbine. Timepoints for blood sampling were as follows, according to the administered form. Oral vinorelbine: 0 h (pre-dose), 1.5 h, 3 h, 6 h, 11 h, and 24 h. Intravenous vinorelbine: 0 h (pre-dose), 3 h, 6 h, 11 h, and 24 h. These sampling times included D-optimal sampling times previously determined [9]. At each sampling timepoint, 3 ml of blood was collected in lithium heparin tubes. In patients receiving intravenous vinorelbine, blood samples were collected from the arm contralateral to the drug infusion arm. Blood samples were immediately frozen at − 20 °C and then stored at − 80 °C until analysis. Blood concentrations of vinorelbine and its metabolite, 4′-*O*-deacetylvinorelbine, were quantified by a validated liquid chromatography–tandem mass spectrometry method [19] at SIMM Bioanalytical Laboratory (Shanghai, China). The lower limit of quantification was 0.25 ng/ml for both compounds.

### Pharmacokinetic data analysis

#### Calculation of individual pharmacokinetic parameters

Empirical Bayes estimates of vinorelbine pharmacokinetic parameters were calculated using a previously developed population pharmacokinetic IV model [9] and an oral model [10] for, respectively, oral and IV data. Briefly, a linear three-compartment model with a zero-order infusion rate for intravenous vinorelbine and first-order absorption rate for oral vinorelbine was used to describe blood vinorelbine concentrations over time. Elimination from the central compartment was modelled with a first-order rate constant. Individual concentration vs. time data were fitted using the post hoc option in NONMEM^®^ version 6.2 (ICON Development Solutions, Ellicott City, MD, USA). The overall goodness of fit between observed and predicted blood vinorelbine concentrations was evaluated through graphical appraisal of individual weighted residuals over time. Individual values of apparent total clearance (Cl_tot_/*F*) for oral vinorelbine and absolute total clearance (Cl_tot_) for intravenous vinorelbine were estimated and summarized by vinorelbine administration route (i.e., oral or intravenous) using descriptive statistics.

As the DVRL metabolite exhibited only very low concentration levels (< 10 ng/ml), no model has been developed for DVRL. Graphically, no major difference between Asian and European population was evidenced (data not shown).

### Comparison between Asian data set and reference European data set

Empirical Bayes estimates of vinorelbine pharmacokinetic parameters from the Asian data set were compared to those from a phase I pharmacokinetic study performed in Europe (reference European data set) [11]. The reference European data set was chosen, as it represented a robust and reliable evaluation of both oral and intravenous vinorelbine pharmacokinetics under strictly controlled and standardized conditions of administration similar to those in the studies of Asian patients. Furthermore, pharmacokinetic assessment from the reference European study was performed using the same bioanalytical method as that used in the present study. Details of the methodology used in the reference European data set have been previously published [11]. Pharmacokinetic parameters investigated included absolute total clearance (Cl_tot_) for intravenous vinorelbine, apparent total clearance (Cl_tot_/*F*) for oral vinorelbine, and inter-individual variability (CV%) in bioavailability and Cl_tot_. Comparison of the pharmacokinetic parameters between the Asian and reference European data set was performed on log-transformed values using one-way analysis of variance. *p* values < 0.05 were considered statistically significant.

### Population pharmacokinetic modelling

To further evaluate the potential effect of ethnicity on the pharmacokinetics of vinorelbine, the Asian data set (oral: *n* = 47; intravenous: *n* = 34) was combined with two large historical vinorelbine pharmacokinetic databases (oral/iv model: *n* = 175 with *n* = 50 iv data and 125 oral; iv model: *n* = 77) built from several phase I and II studies performed in Europe and analyzed using the respective population pharmacokinetics models previously developed for each route of administration [9, 10]. Concentration vs. time data were fitted using the non-linear mixed effect approach with the NONMEM^®^ program using the same estimation method than in the historical model (i.e., FO for oral model and FOCE for iv model). The models were first refined through estimation of a new set of parameters, including the significant covariate effects previously identified as relevant (e.g., body surface area, creatinine clearance as calculated by the Cockcroft and Gault formula, grade of transaminase elevation, and platelet count). Thereafter, ethnicity was tested as a new covariate and integrated into the final population pharmacokinetic model. The potential effect of ethnicity was tested on the typical values of vinorelbine clearance using the intravenous model, and on both clearance and bioavailability using the oral model.

Ethnic covariate was modelled as a power function of the typical pharmacokinetic parameter. For example, the ethnic covariate model for clearance parameter was

$${\text{Cl}}_{ij} = \overline{\text{Cl}} \cdot \theta^{\text{cov}} \cdot e^{{\eta i{\text{Cl}}}} \cdot e^{{\eta j{\text{Cl}}}} ,$$

where Cl_*ij*_ is the clearance (Cl) of individual *i* and occasion *j*, Cl is the typical value of Cl in the population, *η*_*i*Cl_ is the random inter-patient variability, *η*_*j*Cl_ is the random inter-occasion variability, *θ* is the shift parameter describing the systematic dependence of the pharmacokinetic parameter on individual covariate value, and Cov is the individual covariate value [i.e., ethnicity coded either as 0 (European) or 1 (Asian)].

The relevance of a possible effect of the ethnicity was evaluated by several criteria:

1. Objective function value (OFV): a reduction in OFV of more than 3.84 from the reference model (excluding the ethnic covariate) was required to conclude a statistical significance at a nominal α risk of 5% (log-likelihood ratio test).
2. Magnitude of the interaction effect: an effect of more than or equal to 20% was considered to be clinically relevant.
3. Precision of point estimate: the interaction effect and its 95% confidence interval, as computed from the normal asymptotic theory using NONMEM^®^, were considered to be relevant if the 95% confidence interval did not include the null effect.

Correctness of each model (i.e., oral and intravenous models) was assessed by standard goodness of fit plots.

## Results

### Patient characteristics

Vinorelbine pharmacokinetic data from the ABC and NSCLC studies in Asian patients were pooled (oral: *n* = 47; intravenous: *n* = 34) and compared to the European reference data set (oral: *n* = 48; intravenous: *n* = 48). Comparison of patient characteristics between the Asian and European data sets showed lower body surface area and slightly higher creatinine clearance (Cockcroft and Gault formula) in Asian patients (Table 1). Other patient characteristics were similar between the two data sets.

**Table 1:** Patient characteristics in Asian and European data sets

	Asian		European
	Oral (*n* = 47)	Intravenous (*n* = 34)	Oral/intravenous^a^ (*n* = 48)
Gender (male/female)	12/35	9/25	11/37
Age (years)	51 [35–71]	53.5 [31–66]	57.5 [25–71]
Body surface area (m^2^)	1.57 [1.35–1.87]	1.60 [1.30–1.82]	1.71 [1.35–2.11]
Creatinine clearance (ml/min)	97.9 [63.2–252]	96.1 [52.3–179]	79.6 [35.9–127]

Data are presented as number or median [range]

^a^Patients from this reference study [11] were randomized to receive first either 80 mg/m^2^ oral vinorelbine, or 30 mg/m^2^ intravenous vinorelbine in a crossover study design with a 2-week washout period. Hence, all 48 patients from the European data set received both oral and intravenous vinorelbine

### Comparison of vinorelbine pharmacokinetics between Asian and European patients

**Fig. 1:**
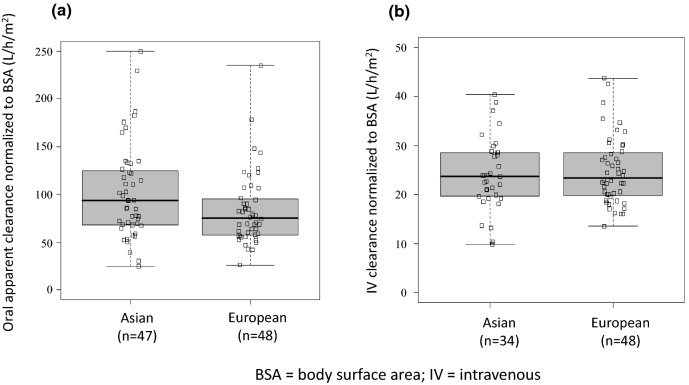
Comparison of **a** apparent total clearance (Cl_tot_/*F*) of oral vinorelbine and **b** absolute total clearance (Cl_tot_) of intravenous vinorelbine between Asian and European patients. *BSA* body surface area, *IV* intravenous

Vinorelbine clearance was comparable between Asian and European patients regardless of the route of administration (Fig. 1). Although there was a slight trend towards a higher oral apparent clearance normalized to body surface area in Asian patients (median [range] 89.4 [25–250] l/h/m^2^) compared with European patients (median [range] 76.7 [26–235] l/h/m^2^), this trend did not reach statistical significance (*p* = 0.07; Fig. 1a). Intravenous vinorelbine clearance in Asian patients (median [range] 23.75 [9.87–40.42] l/h/m^2^) and European patients (median [range] 23.40 [13.55–43.74] l/h/m^2^) was found to be comparable (*p* = 0.57; Fig. 1b).

### Population pharmacokinetic analysis

#### Effect of ethnicity on vinorelbine pharmacokinetics

Pharmacokinetics data from Asian patients were pooled with the historical European reference database for population pharmacokinetics analysis (oral: *n* = 222; intravenous: *n* = 111).

**Fig. 2:**
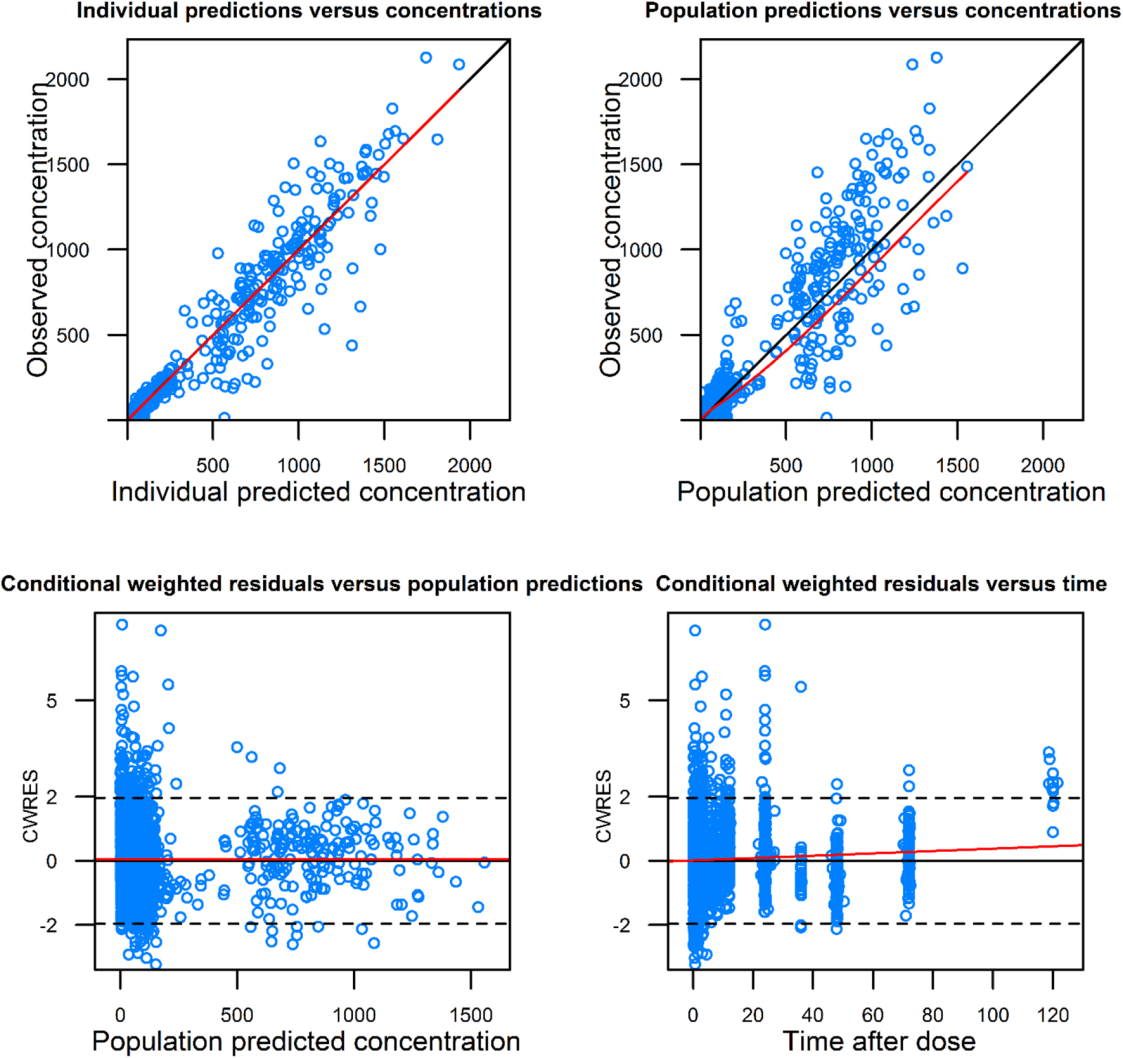
Basic goodness of fit of oral model with ethnicity covariate. Concentrations are expressed as ng/ml; time is expressed as hours

**Fig. 3:**
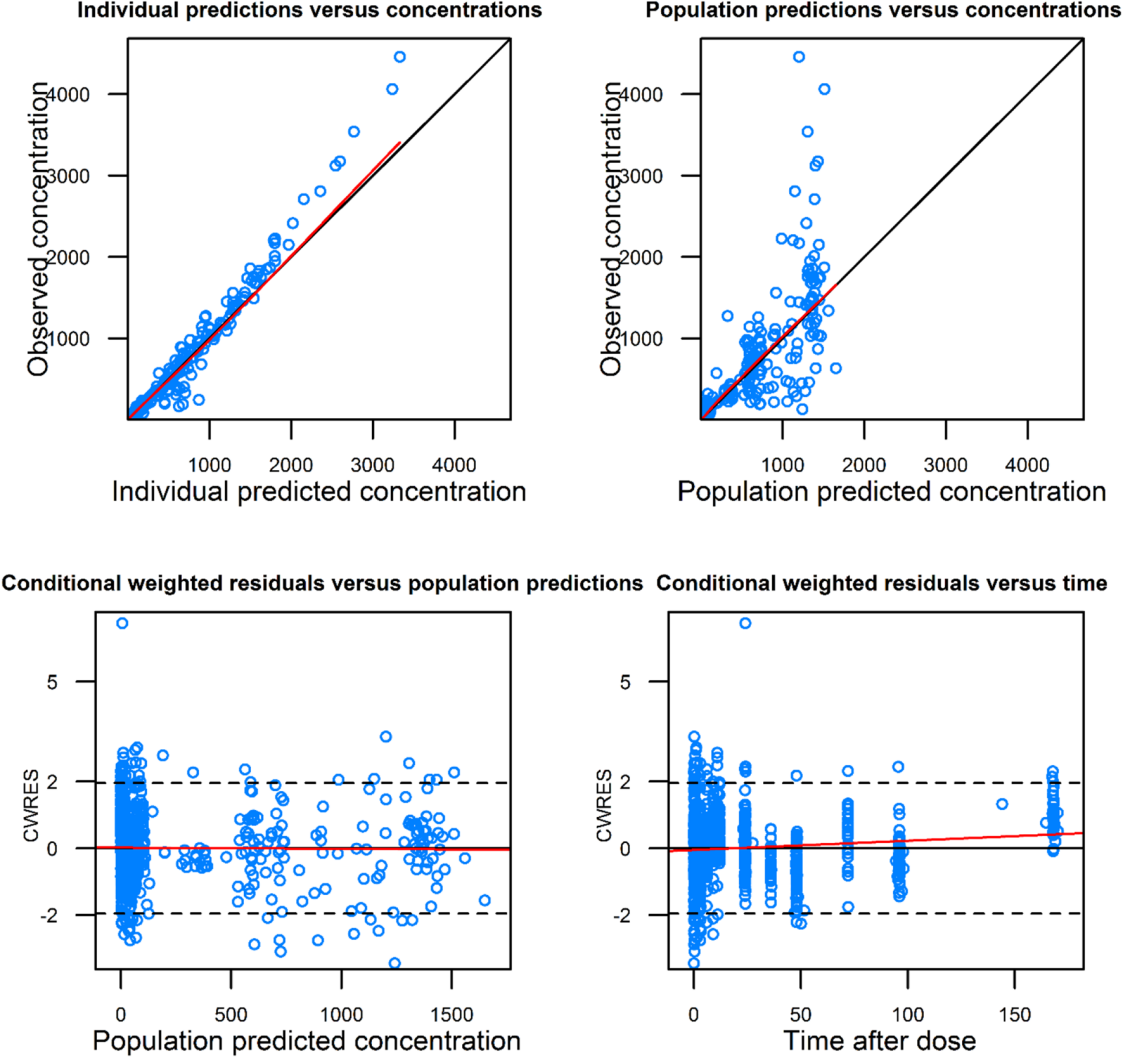
Basic goodness of fit of IV model with ethnicity covariate. Concentrations are expressed as ng/ml; time is expressed as hours

**Fig. 4:**
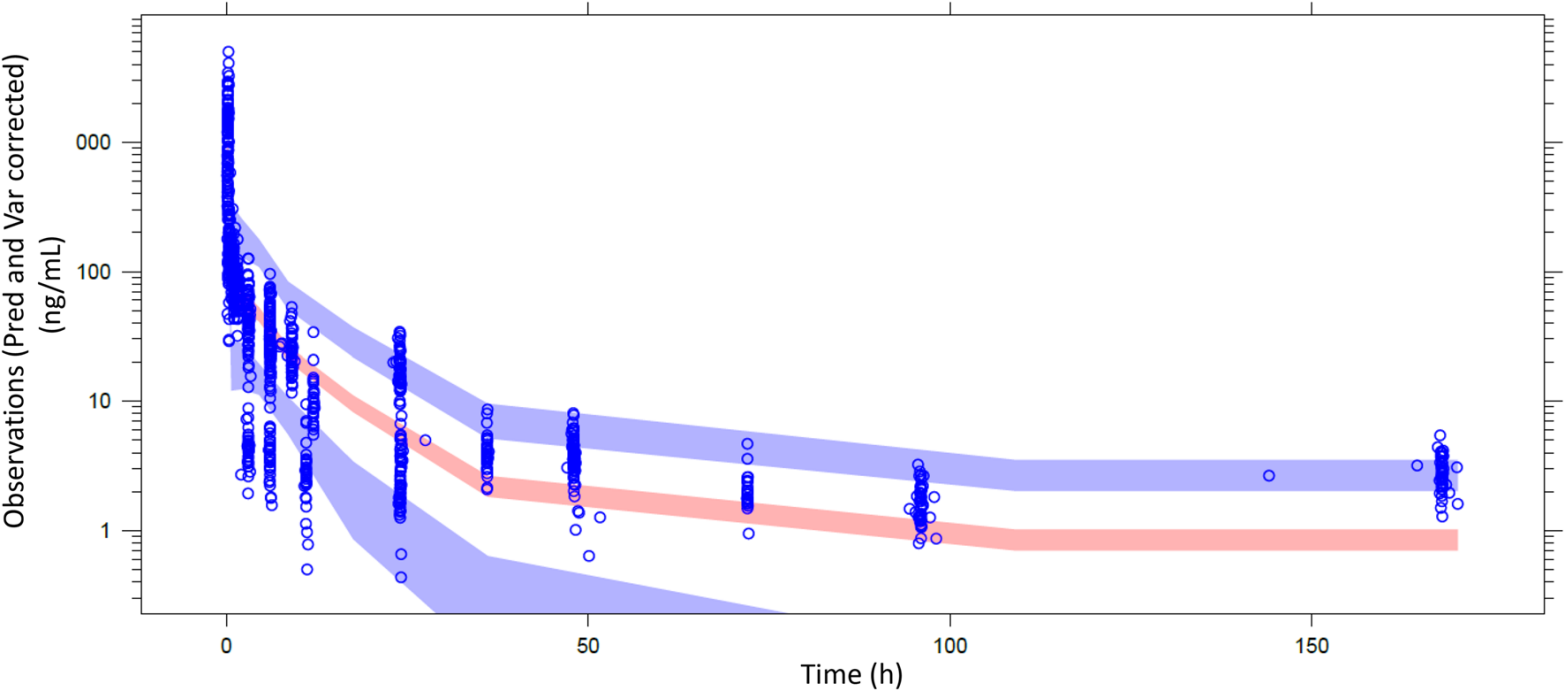
Visual predictive check (IV model). Dot points represent observed values. Areas are, respectively, confidence interval of P5, median and P95 simulated values

**Fig. 5:**
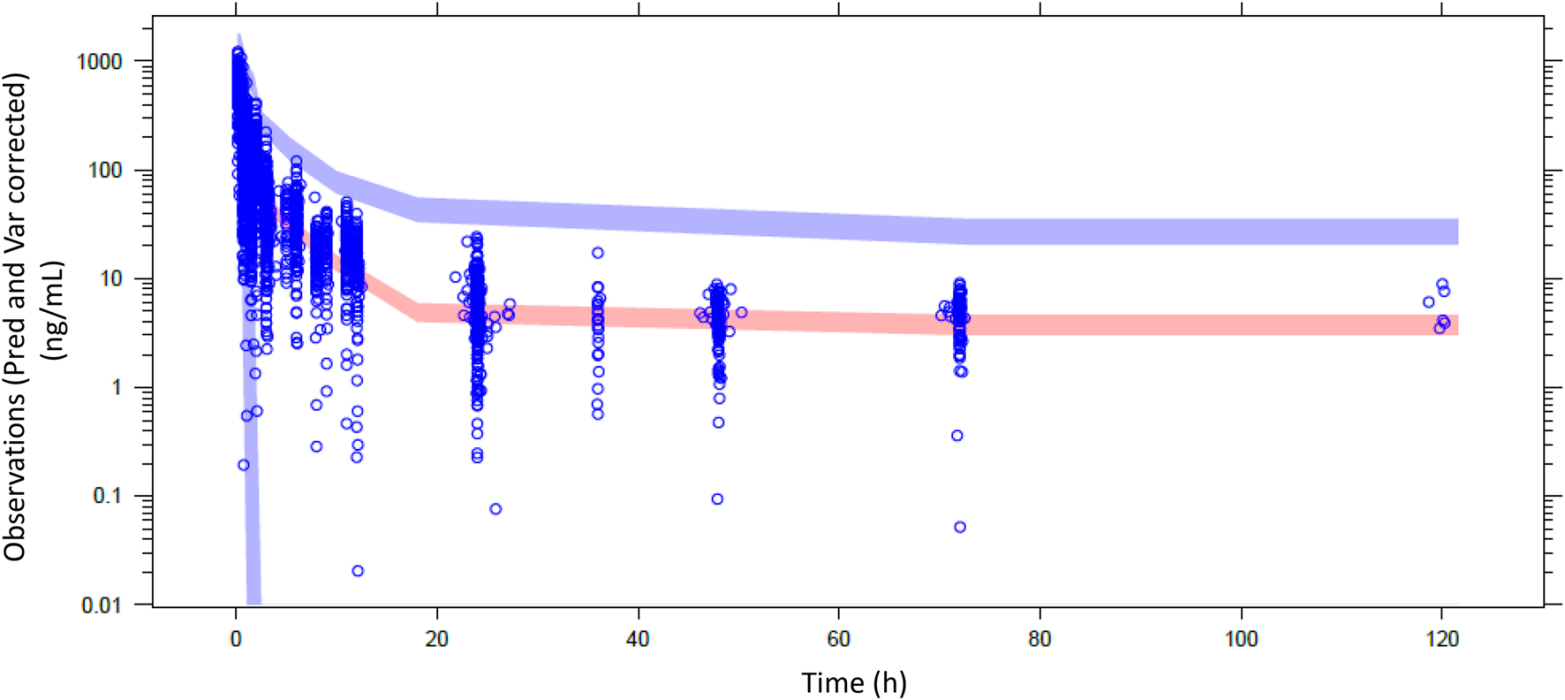
Visual predictive check (oral model). Dot points represent observed values. Areas are, respectively, confidence interval of median and P95 simulated values. Confidence interval of P5 does not appear because of negative values

Population pharmacokinetic analysis was conducted in two steps. First, a “control” model was developed without ethnicity as a covariate and using the same parameterization as historically (i.e., same fixed and random parameters and same residual error model). Descriptive statistics of the covariates are presented in Table 2. Next, ethnicity was introduced as a new covariate to the control model, resulting in a “test” model. Results of the population pharmacokinetic models are presented in Table 3. Goodness of fit of the final models including the ethnicity covariate indicates the model adequacy (Figs. 2, 3). Pred and variance-corrected VPC plots show that observations are included within the range of concentrations simulated with the models (Figs. 4, 5).

**Table 2:** Descriptive statistics of the covariates used in the population pharmacokinetic models

Database covariate	Oral model		IV model	
	Asian	Historical	Asian	Historical
BSA	1.57 [1.35–1.87]	1.72 [1.32–2.33]	1.59 [1.3–1.82]	1.7 [1.35–2.33]
Creatine clearance < 70 ml/min	10.6%	41%		
Platelets	250 [103–659]	267 [90–540]		
Transaminases grade ≥ 1	10.6%	18%

**Table 3:** Estimates from the population pharmacokinetic models for oral and intravenous vinorelbine

	Oral vinorelbine (*n* = 222)		Intravenous vinorelbine (*n* = 111)	
	Control model	Test model (with ethnicity covariate on absolute bioavailability [*F*])	Control model	Test model (with ethnicity covariate on Cl_tot_)
Objective function value (OFV)	17,819.5	17,818.2	5217.4	5216.7
Change in OFV^a^	–	− 1.3	–	− 0.7
Inter-individual variability in Cl_tot_ (CV%)	33.9	33.7	28.1	28.0
Inter-individual variability in Bioavailability (CV%)	20.5	20.6	–	–
Estimated magnitude of ethnic effect (%) [95% CI]	–	5.3 [− 19.0 to 29.6]	–	− 5.1 [− 17.5 to 7]

^a^A decrease from the control model in OFV higher than 3.8 and 7.8 was needed to conclude to any statistically significant difference at 5% and 0.5% risk levels, respectively

For oral vinorelbine, inclusion of ethnicity as a covariate did not significantly improve the fit of the model (change in OFV = − 1.3; Table 2). Inter-individual variability in total clearance and bioavailability changed minimally from 33.90% (control model) to 33.70% (test model) and from 20.50 to 20.60%, respectively. The estimated magnitude of the effect of ethnicity was not significantly different from zero (5.3%; 95% CI from bootstrap results: − 20 to 306%).

Likewise, for intravenous vinorelbine, inclusion of ethnicity as a covariate also did not significantly improve the fit of the model (change in OFV = − 0.7; Table 2). Inter-individual variability in total clearance changed minimally from 28.1% (control model) to 28.0% (test model). The estimated magnitude of the effect of ethnicity was not significantly different from zero (− 5.1%; 95% CI from bootstrap results: − 19.1 to 9.1%).

## Discussion

The bioavailability and elimination of drugs are known to be influenced by ethnicity, especially for drugs which undergo hepatic metabolism [20]. Given that hepatic elimination is the main route of vinorelbine excretion [21], the potential ethnic differences in the pharmacokinetics of vinorelbine must be investigated. The present study compared pharmacokinetics data obtained from Asian patients to those from European patients to evaluate the potential influence of ethnicity on vinorelbine exposure. Vinorelbine clearance was not significantly different between Asian and European patients. Moreover, the population pharmacokinetic analyses give robust results and showed no relevant influence of ethnicity on vinorelbine clearance and bioavailability for both the oral and intravenous routes of administration. Overall, the pharmacokinetics of vinorelbine were found to be comparable between Asian and European patients, and data for the Asian patients were pooled from two-phase II studies conducted in China. Vinorelbine doses administered, blood sampling timepoints, duration of study, and bioanalytical methods used were similar in both studies. Although there were some slight differences in patient gender proportions between studies due to the cancer types studied, they were not expected to influence the pharmacokinetics of vinorelbine. Data for the European patients were obtained from a phase I pharmacokinetic study performed in Europe [11]. This reference European data set was selected for comparison, as it represented a robust and reliable evaluation of both IV and oral vinorelbine pharmacokinetics under strictly controlled and standardized conditions of administration. The intensive sampling schedule (12 timepoints) over a 168 h time period ensured a full description of the PK profile. The European trial also shared similar conditions of administration for both oral and IV vinorelbine with that of the Asian studies: oral vinorelbine was taken with food and with oral 5-HT_3_ antagonist for anti-emetic treatment and IV vinorelbine was infused over a short 10 min period. Moreover, vinorelbine blood concentrations from the Asian studies were measured with the same bioanalytical method (LC/MS–MS method) as that used in the reference European trial; cross validation of the method was performed to ensure consistency in the results (internal data).

Patient characteristics of the Asian and European data sets were mostly comparable, with some expected exceptions—median body surface area of the Asian patients was significantly less than that of the European patients. To account for these differences, vinorelbine clearance values were adjusted for body surface area. Notably, the adjusted values did not differ significantly between the Asian and European data sets. It may be important to note that vinorelbine dosing regimens differed between the Asian and European studies; oral and intravenous vinorelbine doses administered in the European trial were higher than those used in the Asian studies (oral: 80 vs. 60 mg/m^2^; IV: 30 vs. 25 mg/m^2^) [11]. However, since the pharmacokinetics of vinorelbine is linear [8], vinorelbine clearance is not expected to be dependent on the dose administered.

Ethnic differences in drug bioavailability and metabolism are known to cause variability in responses to anticancer drugs [22]. One example is that of vincristine, a vinca alkaloid, which is metabolized more efficiently by CYP3A5 than by CYP3A4 enzymes. Ethnic differences in expression of CYP3A5 between ethnic groups (70% prevalence in African Americans vs. 20% in Caucasians) resulted in significant variability in vincristine-associated neurotoxicity (4.8% vs. 34.8%, respectively) [23]. Caucasians also experienced higher grades of toxicity and were more likely to have their vincristine doses reduced or omitted than African Americans [23]. Based on the comparison between Asian and European data sets, the present analysis suggests comparable vinorelbine clearance between Asian and European patients. Further evaluation by population pharmacokinetic analysis in large historical vinorelbine pharmacokinetic databases showed that ethnicity was not a relevant covariate for absolute bioavailability, as its inclusion into the oral model did not show any significant improvement of fit. There was also no decrease in inter-individual variability when the ethnic covariate was integrated into the model. This is in line with the metabolism knowledge of vinorelbine, mainly involving esterase and CYP3A4 enzymes [24], which are not described as highly functionally polymorphic in the European and Asian populations.

Assuming the absence of co-morbidities, similarities in pharmacokinetic behaviors between Asian and European patients suggest that similar dosage regimen of vinorelbine may be used in both populations. Furthermore, duplication of effort and cost may be avoided as data for either of these populations could be bridged to the other.

In conclusion, the results obtained from the comparison between Asian and European data sets and population pharmacokinetic analysis demonstrated that the pharmacokinetics of oral and IV vinorelbine was not different between Asian and European patients. Knowledge of the pharmacokinetic behavior of vinorelbine in these populations could help to improve individualization of treatment regimens in terms of therapeutic outcomes and safety.
